# Association of IL-10 polymorphisms with hepatitis B virus infection and outcome in Han population

**DOI:** 10.1186/s40001-016-0218-9

**Published:** 2016-06-01

**Authors:** Lei Gao, Xi Chen, Lian Zhang, Di Wu, He Zhao, Junqi Niu

**Affiliations:** Changchun University of Traditional Chinese Medicine, Changchun, 130117 Jilin China; Tumor Center, The 1st Hospital of Jilin University, Changchun, 130021 Jilin China; Ministry of Education Key Laboratory of Zoonosis, The 1st Hospital of Jilin University, No. 71 Xinminda Street, Changchun, 130021 Jilin China

**Keywords:** Interleukin-10, Hepatitis B virus, Single nucleotide polymorphism

## Abstract

**Background:**

This study evaluated the correlation of single nucleotide polymorphisms interleukin (IL)-10-592 and -1082 with hepatitis B virus (HBV) susceptibility and recovery.

**Methods:**

Total 190 chronic hepatitis B (CHB) patients, 81 individuals with self-limited HBV infections and 81 normal controls from the first Hospital of Jilin University were recruited. The IL-10 polymorphisms were detected by polymerase chain reaction with restriction fragment length polymorphism (PCR–RFLP). The *χ*^2^ test (*p* < 0.05) and Fisher’s exact test were separately performed to analyze and compare the genotype frequencies of IL-10-592 and -1082 among different groups. Furthermore, logistic regression analysis (*p* < 0.05) was conducted to determine the correlation of genotypes with HBV infection and recovery. Genotype A/A, A/C and C/C of IL-10-592 had been detected in the three groups.

**Results:**

The frequencies of -592A separately were 55.56, 64.67 and 55.33 % in the three groups. Genotypes of IL-10-592 only had significant difference among the patients and normal controls (*p* = 0.021). Genotypes A/A, A/G and G/G of IL-10-1082 were detected in CHB patients and individuals with self-limited HBV infection; however, genotype G/G had not been detected in normal controls. The frequencies of -1082G separately were 3.68, 6.17 and 11.11 % in the three groups. Genotypes of IL-10-1082 only had no significant difference among the patients and individuals with self-limited HBV infections (*p* = 0.130). We found that their risks of HBV infection existed significant difference.

**Conclusion:**

The IL-10-592 and -1082 polymorphisms might be associated with HBV infection, but not with the recovery after HBV infection.

## Background

Hepatitis B virus (HBV) infection is the main cause for acute and chronic hepatitis. Besides, HBV infection is closely related to the incidence of liver cirrhosis and hepatocellular carcinoma. Particularly, in China, its prevalence is high. Among the adults with the initial infection of HBV, approximately 85 % can recover, and about 10–15 % may turn into chronic hepatitis. And among the patients with chronic hepatitis, approximately 20–30 % would develop into liver cirrhosis. After being infected with HBV, human body can present several different clinical outcomes, which are affected by genetic, immunological, virological and experimental factors [1].

Numerous researches have focused on the relationship between cytokine genetic polymorphisms and the susceptibility to HBV [2–15]. Interleukin-10 (IL-10) is a crucial immunoregulatory cytokine secreted by cells including T regulatory lymphocytes, and activated T helper (Th) 2 cells and macrophages [9]. By directly affecting the production of tumor necrosis factor, IL-10 can take part in the regulation of inflammatory responses [16]. The promoter region contains several components of transcription factors, which is closely related to regulation of gene expression [17, 18], so the single nucleotide polymorphisms (SNPs) of IL-10 promoter affect the expression level of IL-10. Many SNPs in the proximal promoter of IL-10 have been studied to investigate their association with HBV infection, such as polymorphisms on -592 site [19–21], -819 site [10, 11, 20], -872 site [22], and -1082 site [8, 9, 21]. However, polymorphisms on -592 site and -1082 site have not been determined among chronic hepatitis B (CHB) patients, self-limited HBV infection and normal controls in Han population yet.

By comparing the distribution frequency of different genotypes of IL-10-592 site and IL-10-1082 site in CHB patients, individuals with self-limited HBV infections and normal controls, this study attempted to determine whether gene polymorphisms in IL-10-592 and IL-10-1082 have correlation with the susceptibility to HBV and its turnover.

## Patients and methods

### Subjects

A total of 190 CHB patients (144 men and 46 women; average age, 41.55 ± 9.55 years) were from infectious diseases department of the first Hospital of Jilin University, whose diagnosis was based on “The guideline of prevention and treatment for chronic hepatitis B” published by Chinese Society of Hepatology and Chinese Society of Infectious Diseases, Chinese Medical Association in 2005. A total of 81 individuals with self-limited HBV infection cases (60 men and 21 women; average age, 42.51 ± 11.30 years) were from the target groups of epidemiological investigation by infectious diseases department, which were defined as patients with no history of hepatitis B, with normal liver function, whose HBV serum markers showed hepatitis B surface antibody (HBsAb) and hepatitis B core antibody (HBcAb) positive while hepatitis B surface antigen (HbsAg) and hepatitis Be antigen (HBeAg) negative. And a total of 81 normal control cases (58 men and 23 women; average age, 41.59 ± 11.79 years) were also from the target groups of epidemiological investigation, in which people were healthy according to medical examination. Furthermore, all the participants were Han people in northern China. This project was approved by the ethical committee of the first Hospital of Jilin University (the reference number: 2014-224 and 2014-225) and informed consents were signed by all participants before study initiation.

### Specimen collection

Fasting blood taken in the early morning was added into the test tube with EDTA-K_2_ anticoagulant and reserved after fully mixed. Genomic DNA was extracted from peripheral blood leucocytes using DNA extraction kit (Promega, Wisconsin, USA), and then stored at −20 °C.

### Genotyping

Two SNPs of IL-10 (IL-10-592 and IL-10-1082) were detected and genotyped using polymerase chain reaction with restriction fragment length polymorphism (PCR–RFLP). Their corresponding primers (Sangon, Shanghai, China) were designed using the standard sequences rs1800872 and rs1800896 from SNPs database website as templates. The primers used for rs1800872 included the forward primer: 5′-GGTGAGCACTACCTGACTAGC-3′ and the reverse primer: 5′-TAGGTCACAGTGACGTGG-3′. The primers used for rs1800896 included the forward primer: 5′-CTCGCCGCAACCCAACTGGC-3′ and the reverse primer: 5′-GTAAGGGACCTCCTATCCAG-3′. PCR was conducted in a volume of 50 μL with 25 μL 2× Taq Plus PCR MasterMix (TIANGEN, Beijing, China), and concentration of each primer was 20 pmol/μL. PCR was carried out under the conditions: 94 °C for 5 min, 30 cycles for 94 °C 45 s, 61 °C 45 s, 72 °C 1 min, and 72 °C for 10 min. RFLP digestion system was carried out in a volume of 20 μL with 0.1–0.5 μg the PCR product, 0.5 μL (5 u/μL) *Mnl*I restriction enzyme, 2 μL 10× NEB buffer, 0.2 μL 100× BSA, 17.3 μL sterilized double-distilled water. The digested condition was 37 °C for 4 h. We used 2.0 μg digestion products to perform agarose gel electrophoresis and then observed the results under the gel imaging system (TANON, Shenyang, China).

### Statistical analysis

Statistical tests including the χ^2^ test and Fisher’s exact test were conducted using the SPSS 11.5 software. The Hardy–Weinberg (H–W) equilibrium and frequencies of the genotypes among different groups were compared. The *χ*^2^ tests were performed to analyze the genotype frequency of IL-10-1082 G/A among different groups. The 95 % confidence interval (95 % CI) and the odds ratio (OR) were calculated. Logistic regression analysis was also used to determine the correlation of a specific genotype with HBV infection and recovery. *p* < 0.05 was considered as statistically significant.

## Results

### Three genotypes of IL-10-592 and IL-10-1082 were detected in Han population

**Fig. 1:**
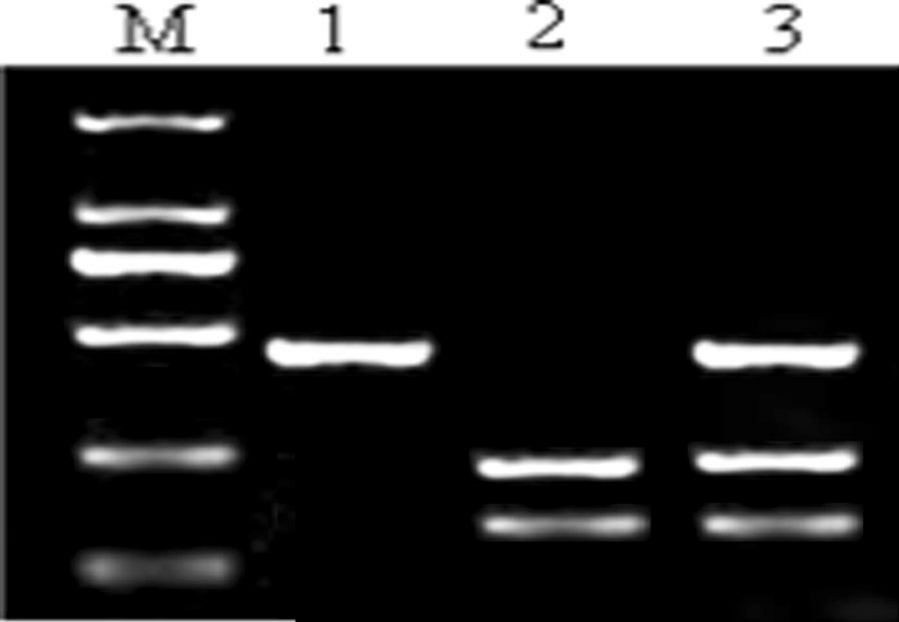
Electrophoresis result of IL-10-592 polymorphisms. M: DL2000 DNA marker, 100, 250, 500, 750, 1000, 2000 bp from *bottom* to *top* of the gel; *lane 1* genotype C/C; *lane 2* genotype A/A; *lane 3* genotype A/C

The 410-bp sequences in IL-10-592 promoter were amplified by normal PCR and analyzed by RFLP. Three RFLP patterns appeared after digestion with the restriction enzyme *Rsa*I (Fig. 1). The band in lane 1 was 410 bp and its genotype was C/C. The genotype of the two bands of 234 and 176 bp observed in lane 2 was A/A. The bands in lane 3 separately were 410, 234 and 176 bp, and their genotype was A/C. Overall, we found three IL-10-592 genotypes A/A, A/C and C/C in Han population of northern China.

**Fig. 2:**
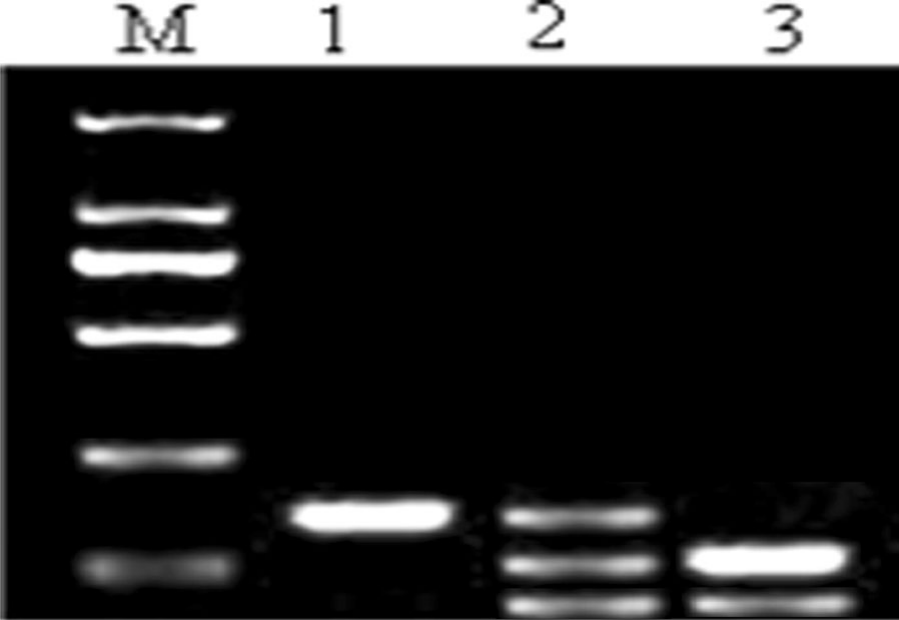
Electrophoresis result of IL-10-1082 polymorphisms. M: DL2000 DNA marker, 100, 250, 500, 750, 1000, 2000 bp from *bottom* to *top* of the gel; *lane 1* genotype A/A; *lane 2* genotype A/G; *lane 3* genotype G/G

The 159-bp sequences in IL-10-1082 promoter were amplified by normal PCR and analyzed by RFLP. Three RFLP patterns appeared after digestion with the restriction enzyme *Mnl*I (Fig. 2). The band in lane 1 was 159 bp and its genotype was A/A. The genotype of the three bands of 159, 103 and 56 bp observed in lane 2 was A/G. And, the bands in lane 3 were 103 and 56 bp, and their genotype was G/G. In general, this study found three genotypes A/A, A/G and G/G in Han population of northern China.

### Distribution frequencies of IL-10-592 and IL-10-1082 genotypes

We analyzed the distribution frequencies of different genotypes of IL-10-592 and IL-10-1082 among CHB patients, individuals with self-limited HBV infections and normal controls (Table 1). The genotype frequencies of IL-10-592 and IL-10-1082 among these three groups all met the H–W equilibrium. The genotype frequencies of A/A, A/C and C/C on -592 site were 25.56, 60.00 and 14.44 % in CHB patients, 40.00, 49.33 and 10.67 % in individuals with self-limited HBV infections, and 34.67, 41.33 and 24.00 % in normal controls, respectively. Thus, the frequencies of -592A separately were 55.56, 64.67 and 55.33 % in CHB patients, individuals with self-limited HBV infections and normal controls. The genotype frequencies of G/G, A/G and A/A on -1082 site were 0.53, 6.32 and 93.16 % in CHB patients; 3.70, 4.94 and 91.36 % in individuals with self-limited HBV infections; and 0.00, 22.22 and 77.78 % in normal controls, respectively. Therefore, the frequencies of -1082G were 3.68, 6.17 and 11.11 % in CHB patients, individuals with self-limited HBV infections and normal controls, respectively. Then, *χ*^2^ tests were performed.

**Table 1:** Genotype frequencies of IL-10-592 and IL-10-1082 in the three groups

SNPs	Genotype No. (%)	Group		
		Patients group	Self-limited infection group	Normal group
IL-10-592	A/A	46 (25.56)	30 (40.00)	26 (34.67)
	A/C	108 (60.00)	37 (49.33)	31 (41.33)
	C/C	26 (14.44)	8 (10.67)	18 (24.00)
IL-10-1082	G/G	1 (0.53)	3 (3.70)	0 (0.00)
	A/G	12 (6.32)	4 (4.94)	18 (22.22)
	A/A	177 (93.16)	74 (91.36)	63 (77.78)

Genotype A/A, A/C and C/C of IL-10-592 had been detected in the three groups. According to continuity correction *χ*^2^ tests, genotype C/C frequency increased significantly (*χ*^2^ = 7.742, *p* = 0.021) in normal controls compared with CHB patients. Furthermore, genotype frequencies of individuals with self-limited HBV infections versus normal controls (*χ*^2^ = 4.661, *p* = 0.097), and CHB patients versus individuals with self-limited HBV infections (*χ*^2^ = 5.332, *p* = 0.070) had no significant difference.

Genotype A/A of IL-10-1082 was the highest frequency in all of the participants, while genotypes A/G and G/G were relatively rare. Particularly, in the normal controls, genotype G/G had not been detected. By continuity correction *χ*^2^, we compared genotype frequencies of CHB patients with normal controls. In patients, genotype A/A frequency increased significantly, while A/G frequency reduced (*χ*^2^ = 13.883, *p* = 0.001). Besides, by continuity correction *χ*^2^, we compared that of individuals with self-limited HBV infections with normal controls. In individuals with self-limited HBV infections, genotype A/A frequency increased significantly, while A/G frequency reduced (*χ*^2^ = 14.679, *p* = 0.001). Furthermore, by the Fisher exact probabilistic method, we compared genotype frequencies of CHB patients with individuals with self-limited HBV infections. Different genotype frequencies between the two groups had no significant difference (*p* = 0.130).

### Association of IL-10-592 and IL-10-1082 polymorphisms with HBV infection

Using logistic regression, the association of IL-10-592 genotypes with HBV infection among CHB patients and normal controls were compared (Table 2). The risk of HBV infection of genotype A/C carriers was 2.412 times of the genotype C/C carriers (OR = 2.412, 95 % CI 1.172–4.963). Thus, the risk of HBV infection had significant difference between genotype A/C carriers with genotype C/C carriers (*p* < 0.05).

**Table 2:** The risk analysis of IL-10-592 and IL-10-1082 polymorphisms associated with HBV infection (the patients versus the normal controls)

SNPs	Genotype	OR	95 % CI	*p*
IL-10-592	C/C	1	–	–
	A/A	1.225	0.567–0.502	2.645
	A/C	2.412	1.172–4.963	0.017*
	A allele	1.870	0.954–3.668	0.068
IL-10-1082	A/A	1	–	–
	A/G	0.237	0.108–0.520	0.00*
	G allele	0.257	0.119–0.555	0.001*

*SNP* single nucleotide polymorphism, *OR* odds ratio, *95* *% CI*, 95 % confidence interval, *A allele* A/A + A/C, *G allele* A/G + G/G

* *p* < 0.05

We compared the association of IL-10-1082 genotypes with HBV infection among CHB patients and normal controls using logistic regression (Table 2). The risk of HBV infection had significant difference between genotype A/G carriers with genotype A/A carriers (*p* < 0.05). In detail, the risk of HBV infection of genotype A/G carriers was only 0.237 times of the genotype A/A carriers (OR = 0.237, 95 % CI 0.108–0.520), while that of G allele carriers (A/G + G/G) was 0.257 times of the homozygous AA-type carriers (OR = 0.257, 95 % CI 0.119–0.555).

Afterward, we compared the association of IL-10-1082 genotypes with HBV infection among self-limited HBV infections and normal controls using logistic regression (Table 3). In detail, the risk of HBV infection of genotype A/G carriers was only 0.189 times of the genotype A/A carriers (OR = 0.189, 95 % CI 0.061–0.588), while that of G allele carriers (A/G + G/G) was 0.331 times of the homozygous AA-type carriers (OR = 0.331, 95 % CI 0.130–0.844). Therefore, the risk of HBV infection had significant difference between genotype A/G carriers with genotype A/A carriers (*p* < 0.05).

**Table 3:** The risk analysis of IL-10-1082 polymorphisms associated with HBV infection (individuals with self-limited infections versus the normal controls)

SNP	OR	95 % CI	*p*
A/A	1	–	–
A/G	0.189	0.061–0.588	0.004*
G allele	0.331	0.130–0.844	0.021*

*SNP* single nucleotide polymorphism, *OR* odds ratio, *95* *% CI* 95 % confidence interval, *G allele* A/G + G/G

* *p* < 0.05

## Discussion

In this study, we identified the polymorphisms of -592 and -1082 in the IL-10 promoter region and the correlation of these polymorphisms with HBV susceptibility and recovery in Han population. Genotype A/A, A/C and C/C of IL-10-592 had been detected in the three groups. Similarly, genotypes A/A, A/G and G/G of IL-10-1082 were found in patients and individuals with self-limited HBV infections, while genotype G/G was not observed in normal controls.

After HBV infection, the immune response can be induced to eliminate the virus. As a crucial Th-2 cell cytokine, IL-10, whose expression can be effected by the polymorphisms in the regulatory regions, plays an important role in immune responses [23]. Crawley et al. [24] found that IL-10-1082, -819, -592 could link to different haplotypes. Haplotype carrying the homozygous ATA was with low level of IL-10 while haplotype carrying the homozygous GCC was with high level of IL-10. Meanwhile, the ACC (-1082, -819 and -592) haplotype could improve individuals’ anti-hepatitis B surface antigen (anti-HBs) antibody production almost twice of individuals without this haplotype, while -1082A allele could suppress individuals’ anti-hepatitis A virus (anti-HAV) antibody production in comparison to individuals homozygous for the -1082G allele homozy [11].

This study found that -592G mutated into -592A could enhance the susceptibility to HBV, while -1082A mutated into -1082G could reduce the susceptibility to HBV. Thus, -1082G allele was the protective factor against HBV infection, and the SNPs of IL-10 at -592 and -1082 sites were associated with HBV infection. This was in accordance with the results of Cheong et al. [10], Shin et al. [25] and Miyazoe et al. [2]. In contrary, Gao et al. [9] found that both IL-10-1082 A/G alleles and IL-10-1082 AA/AG genotypes did not have significant difference between patients with HBV-infected individuals and controls. By conducting meta-analysis, Lu et al. [26] demonstrated that IL-10-1082 A/G alleles have no relation to HBV infection in the Asian population. Wu et al. [27] found that the polymorphisms of IL-10-1082 G/G genotype have correlation with lower HBV viral load and earlier HBeAg seroconversion. Zhang et al. [28] deemed that the SNPs of IL-10 promoter have no correlation with the susceptibility to HBV and DNA replication of HBV after infection, but the SNPs of IL-10 at -819 and -592 sites had association with liver inflammation.

Various regulatory and proinflammatory cytokines, such as IL-10, interleukin-12 (IL-12) and interferon-γ (IFN-γ), can change the course of chronic HBV infection, but their effects on HBV recovery are not entirely clear [8, 29–31]. We found that genotypes of IL-10-592 only had significant difference among the patients and normal controls (*p* = 0.021). Genotypes of IL-10-1082 had significant difference in the patients versus normal controls (*p* = 0.001) and individuals with self-limited HBV infections versus normal controls (*p* = 0.001), but not among the patients and individuals with self-limited HBV infections (*p* = 0.130). So we could conclude that compared with the normal controls, the patients and the individuals with self-limited HBV infections were more susceptible to HBV. Thus, the SNPs of IL-10 at -592 and -1082 sites had no correlation with HBV recovery. And this was in accordance with the results of Sofian et al. [20]. However, the *G* allele at IL-12β SNP rs3212217 (IL-12β-10993) and A allele at IL-10 SNP rs1800872 (IL-10-592) have been reported to predict greater peak alanine aminotransferase (ALT) levels in the immune-clearance phase, and also predict spontaneous HBsAg seroconversion/HBV recovery [32]. These conflicting findings might be resulted by influence of other genes, numbers of participants, characteristics of patients, and many other geographical or epidemiological factors. In the present study, the number and characteristics of participants were limited, and confounding factors were not determined. Considering these limitations, further studies should be done to confirm our findings.

## Conclusion

In conclusion, the polymorphisms of IL-10-592 and IL-10-1082 might be associated with the susceptibility to HBV in Chinese Han population. However, the polymorphisms at -592 and -1082 sites might have no correlation with the turnover after HBV infection.
